# Use of magnetic resonance imaging in assessment of constrictive pericarditis: a Moroccan center experience

**DOI:** 10.1186/1755-7682-4-36

**Published:** 2011-10-19

**Authors:** Amal Lachhab, Nawal Doghmi, Abdellah Zouhairi, Anis Seghrouchni, Fouad Amal Wahid, Abdellatif Boulahya, Wajih Maazouzi, Youssef Elfakir, Omar Taoussi, Rachida Amri, Loubna Belhaj, Laila Haddour, Rhizlane Cherradi, Latifa Oukerraj, Mohamed Cherti

**Affiliations:** 1 Department of Cardiology B, Maternity Hospital, Rabat, Morocco; 2 Department of Cardiovascular Surgery, Ibn Sina Hospital, Rabat, Morocco; 3 Department of Cardiovascular surgery, Military Hospital, Rabat, Morocco; 4 Department of Radiology, Agdal Clinic, Rabat, Morocco

**Keywords:** Constrictive pericarditis, magnetic resonance imaging, pericardial thickening, tagging, adherences

## Abstract

**Background:**

The diagnosis of constrictive pericarditis continues to be a clinical challenge. Magnetic resonance imaging provides excellent visualization of the pericardium. The aim of our study is to clarify the contribution of this non invasive exploration in the diagnosis of constrictive pericarditis in our center.

**Methods:**

we conducted a prospective study over a period of two years, since 2008, covering a series of patients (n = 11), mean age 44 ± 15 years, in whom constrictive pericarditis was suspected clinically and on transthoracic echocardiography. We studied its characteristics on magnetic resonance imaging.

**Results:**

Magnetic resonance imaging confirmed the diagnosis showing pericardial thickening in all cases, measuring 8.2 +/- 2.6 mm on average, circumferential in 64%, and localized in 36%. The imaging data, particularly pericardial thickening and its topography, were confirmed by surgical exploration, and results were concordant in all cases.

**Conclusion:**

Magnetic resonance imaging is a powerful tool to establish constrictive pericarditis diagnosis.

## Background

Constrictive pericarditis is a rare condition which occurs when a thick, inelastic pericardium encases the heart and restricts expansion, resulting in chronic biventricular diastolic dysfunction, predominant right heart failure, and low systemic output [1]. Although many diagnostic criteria for constrictive pericarditis have been proposed, this diagnosis continues to be a clinical challenge [2]. Traditionally, echocardiography has been widely used for the assessment of constrictive pericarditis. However, this imaging modality can be limited by poor acoustic windows and is often unable to differentiate constrictive pericarditis from restrictive cardiomyopathy. Currently, magnetic resonance imaging (MRI), which provides an excellent visualization of the pericardium, is being increasingly employed to evaluate pericardial diseases, especially constrictive pericarditis. The aim of this study is to report our experience concerning the contribution of MRI in the diagnosis of constrictive pericarditis.

## Methods

From 2008 to 2010, we prospectively included patients referred to our center to investigate suspected constrictive pericarditis. Information regarding clinical and echocardiographic data was obtained from referring physicians. Those patients underwent MRI on a 1.5-Tesla magnetic resonance (Siemens) with breath-holding and free-breathing respiratory gated imaging and with electrocardiographic triggering. The MRI examinations began with the acquisition of survey images in three orthogonal planes: transverse, coronal, and sagittal. Cine MRI images were acquired using true-fisp sequences. Tagging imaging sequences were performed looking for pericardial adherence. Morphological heart study was realized with spin echo (HASTE) sequences. Five to ten minutes after the injection of gadolinium, images were obtained using a phase-sensitive inversion recovery (PSIR) spoiled gradient echo sequence in order to detect late gadolinium enhancement (LGE). Images were acquired in two-chamber, four-chamber, and short-axis planes. All images were analyzed using Argus post-processing software. Maximal pericardial thickness was measured in end-diastole. Pericardial thickness of 4 mm or more was considered pathological.

## Results

**Figure 1:**
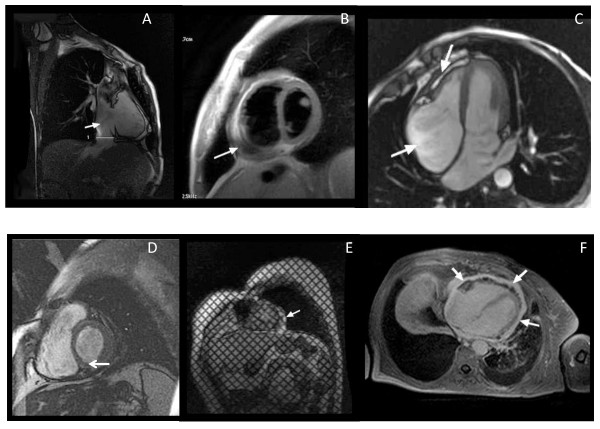
**MR appearences of constrictive pericarditis**. A: Right ventricular vertical long-axis image showing circumferential pericardial thickening, enlarged inferior vena cava; B: short axis image showing circumferential pericardial thickening, encysted pericardial effusion. C: four chamber image showing focal pericardial thickening in front of the right ventricle lateral wall, encysted pericardial effusion, enlarged right atrium; D: short axis image showing focal pericardial thickening in front of the left ventricular inferior and lateral wall. E: short axis tagging image showing focal pericardial thickening and adherence in front of the left ventricular lateral wall. F: four chamber late gadolinium enhancement image showing enhancing pericardium.

During a period of two years, eleven (n = 11) patients underwent MRI for suspected constrictive pericarditis. The mean age was 44 ± 15 years. MRI provided the diagnostic confirmation of constrictive pericarditis in all patients. The youngest patient was only 21 years old and presented with an idiopathic pericarditis; the oldest was 70 years old and had a postsurgical form, occurring after a coronary artery bypass surgery. The sex ratio was (1.2), with six men (54.5%) and five women (45.5%). In our population, four patients (36%) had pericardial tuberculosis and one patient (9%) presented a postsurgical form. Constrictive pericarditis remained idiopathic in six patients (54.5%). All patients were symptomatic, presenting with variable degrees of dyspnea and right heart failure symptoms. On echocardiographic data, thick pericardium was suspected in five (45.5%) cases, biatrial enlargement was observed in six (54.5%) patients and enlargement of the inferior vena cava was seen in the whole study group. In nine (82%) patients, significant inspiratory decrease of transmitral inflow has been registered. Pericardial effusion was observed in two cases. One patient had a concomitant ostium secondum atrial septal defect. MRI data is summarized in Table 1. When analyzing these results, pericardial thickness exceeded 4 mm in all patients. The mean value was 8.2 mm ± 2.6, ranging from 5 mm to 13 mm. Pericardial thickening was circumferential in seven cases (64%) and focal in the remaining ones. Associated pericardial effusion was observed in two patients (18%). MRI also revealed morphological abnormalities with right atrial dilatation in nine patients (82%), left atrial enlargement in eight cases (73%) and an enlarged inferior vena cava in all individuals. In the whole group, cine cardiac evaluation in the short axis plane revealed early diastolic flattening of the interventricular septum with return of normal septal convexity toward the right ventricle during systole. Tagged-cine imaging showed, in eight cases (73%), an adhesion of the thickened pericardium to the myocardium, indicated by the persistent concordance of tagged signals between the pericardium and the myocardium throughout the diastolic and systolic phases. LGE sequences demonstrated an enhancing pericardium in three cases (27%). Figures 1 and2 illustrate examples of MRI images in our patients. Eight (73%) patients underwent surgery: subtotal pericardiectomy through median sternotomy in seven cases; pericardial drainage and biopsy in one patient presenting with an active tuberculosis. One patient had also surgical closure of the atrial defect. During surgery, direct inspection of the pericardium confirmed pericardial thickening in the whole study group. The four patients presenting with tuberculosis pericarditis also received anti-tuberculosis chemotherapy during a twelve-month period. The operative mortality was nil. The three remaining patients have not consented to surgery.

**Table 1:** MRI characteristics of study population

Patient	Age (years)	Sex	Etiologie	Pericardium			Heart chambers					Adhérence (Tagging)	LGE
		
				thickness	Extent	Effusion	IVC	RA	RV	LA	LV		
1	52	F	Tuberculous	8 mm	C	0	E	N	N	N	N	+	+

2	21	M	Idiopathic	8 mm	F	0	E	E	E	E	N	+	0

3	25	M	Idiopathic	7 mm	F	0	E	E	E	N	N	+	0

4	50	M	Idiopathic	13 mm	C	0	E	E	N	E	N	0	0

5	42	F	Tuberculous	12 mm	F	0	E	E	N	E	N	+	0

6	61	M	Idiopathic	8 mm	C	0	E	N	N	N	N	+	+

7	46	F	Idiopathic	12 mm	C	0	E	E	N	E	N	0	0

8	70	M	post-surgical	6 mm	F	0	E	E	N	E	N	+	+

9	24	F	Idiopathic	10 mm	C	+	E	E	N	E	N	+	0

10	53	F	Tuberculous	6 mm	C	0	E	E	N	E	N	+	0

11	40	M	Tuberculous	5 mm	C	+	E	E	N	E	N	0	0

F: fe male; M: m ale; C: circumferentia l; F: focal; 0: absent; +: p resent; E: e nlarged; N: no t enlarged; IVC: in ferior v ena cava; RA: right atrium; RV: right ventricular; LA: left atrium; LV: left ventricular; LGE: late gadolinium enhancement.

**Figure 2:**
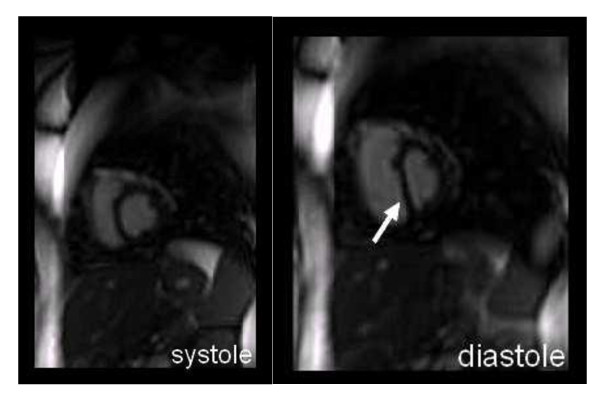
**short axis MRI showing early diastolic flattening of the interventricular septum**.

## Discussion

Constrictive pericarditis is a rare but serious illness, which continues to pose a diagnostic dilemma [2]. It's defined as impedance to diastolic filling caused by a fibrotic pericardium [3]. Several possible etiologies of constrictive pericarditis have been described. Our results support the finding that this condition remains most commonly idiopathic and that tuberculosis is still the predominant cause in developing and underdeveloped countries (36% of patients in our series). In the developed world, cardiac surgery and mediastinal irradiation are quickly becoming the leading causes of constrictive pericarditis [4]. The main problem with this disease is the difficulty to distinguish constrictive from restrictive cardiomyopathy. This differentiation remains a complex but important clinical challenge. In fact, constriction is potentially correctable with pericardiectomy, whereas in restrictive cardiomyopathy, treatment is largely palliative and prognosis is poor [5]. As shown in our study, and as described in many reports, echocardiography is unable to confirm the diagnosis of pericardial constriction in some cases, especially in patients with poor acoustic windows or in localized or obscure forms of constrictive pericarditis [2]. Recently, the development of innovative non-invasive imaging techniques, especially MRI, has substantially helped in the diagnosis of constrictive pericarditis. Advantages of MRI include its potential for tissue characterization, the absence of ionizing radiation, its noninvasiveness, and the ability to scan in any plane. Disadvantages include its difficulty in identifying calcifications because calcium produces no MR signal, and the need for cardiac gating, which may result in inadequate examinations in patients with arrhythmias [6]. Actually, the 2009 SFC/SFR guidelines mention that MRI is a Class I indication in the diagnosis of constrictive pericarditis [7]. The American College of Cardiology Foundation considers MRI as appropriate diagnostic criteria in pericardial constriction [8]. As shown by our study, MRI findings center on the demonstration of thickened pericardium (>4 mm) with secondary signs of constriction, including distorted ventricles (conical and tubular), sigmoid-shaped interventricular septum, large atria and large inferior vena cava [9-11]. Excellent overall sensitivity (88%), specificity (100%), and accuracy (93%) have been reported for MRI [12]. Importantly, increased pericardial thickening may not always imply constriction, and conversely, normal pericardial thickness does not exclude constrictive pericarditis [13]. Unfortunately, in our series, no patient fell within these categories. Furthermore, tagged cine MRI sequence analysis is believed to be well suited for optimal functional imaging in constrictive pericarditis. Tagging images evaluates the adherence and immobility of the pericardial-myocardial interface [14]. Lastly, LGE sequences can reveal enhancing pericardium, suggestive of pericardial inflammation but nonspecific to constrictive pericarditis [2,15].

## Conclusion

Despite the small sample size of our study, which can be explained by the higher cost of MRI, our data supports the concept that MRI is a key tool in the management of patients with suspected constrictive pericarditis.

## List of Abreviations

LGE: late gadolinium enhancement; MRI: magnetic resonance imaging.

## Competing interests

The authors declare that they have no competing interests.

## Authors' contributions

AL and ND were involved in cardiovascular magnetic resonance imaging and analyzing images, in acquisition, analysis and interpretation of the data, and in drafting of the manuscript.

AZ was involved in analyzing and interpretation of the data, and in drafting the manuscript.

AS, FAW, AB and WM were involved in operating patients and in describing surgical findings.

YE and OT were involved in cardiovascular magnetic resonance imaging.

RA, LB, LH, RC, LO and MC were involved in echocardiography's examination of the patients. MC was involved also in revising the manuscript.

All authors read and approved the final manuscript.
